# Preliminary evaluation of alpha-emitting radioembolization in animal models of hepatocellular carcinoma

**DOI:** 10.1371/journal.pone.0261982

**Published:** 2022-01-21

**Authors:** Yong Du, Angel Cortez, Anders Josefsson, Mohammadreza Zarisfi, Rebecca Krimins, Eleni Liapi, Jessie R. Nedrow

**Affiliations:** 1 Russell H. Morgan Department of Radiology & Radiological Science, Johns Hopkins University School of Medicine, Baltimore, MD, United States of America; 2 Department of Radiology, University of Pittsburgh School of Medicine, Pittsburgh, PA, United States of America; Texas A&M University, UNITED STATES

## Abstract

Hepatocellular carcinoma is the most common primary liver cancer and the fifth most frequently diagnosed cancer worldwide. Most patients with advanced disease are offered non-surgical palliative treatment options. This work explores the first alpha-particle emitting radioembolization for the treatment and monitoring of hepatic tumors. Furthermore, this works demonstrates the first *in vivo* simultaneous multiple-radionuclide SPECT-images of the complex decay chain of an [^225^Ac]Ac-labeled agent using a clinical SPECT system to monitor the temporal distribution. A DOTA chelator was modified with a lipophilic moiety and radiolabeled with the α-particle emitter Actinium-225. The resulting agent, [^225^Ac]Ac-DOTA-TDA, was emulsified in ethiodized oil and evaluated *in vivo* in mouse model and the VX2 rabbit technical model of liver cancer. SPECT imaging was performed to monitor distribution of the TAT agent and the free daughters. The [^225^Ac]Ac-DOTA-TDA emulsion was shown to retain within the HEP2G tumors and VX2 tumor, with minimal uptake within normal tissue. In the mouse model, significant improvements in overall survival were observed. SPECT-imaging was able to distinguish between the Actinium-225 agent (Francium-221) and the loss of the longer lived daughter, Bismuth-213. An α-particle emitting TARE agent is capable of targeting liver tumors with minimal accumulation in normal tissue, providing a potential therapeutic agent for the treatment of hepatocellular carcinoma as well as a variety of hepatic tumors. In addition, SPECT-imaging presented here supports the further development of imaging methodology and protocols that can be incorporated into the clinic to monitor Actinium-225-labeled agents.

## Introduction

Hepatocellular carcinoma (HCC) is the most common primary liver cancer and the fifth most frequently diagnosed cancer worldwide, as well as the second most frequent cause of cancer death [1–5]. Most patients with advanced disease are offered only non-surgical palliative treatment options, including systemic chemotherapy, radiofrequency ablation, or intra-arterial therapies [5]. Hepatic tumors derive their blood supply primarily from the hepatic artery, whereas normal liver is mainly fed by the portal vein. Image-guided intra-arterial therapies, such as transarterial embolization (TAE), chemoembolization (TACE) and radioembolization (TARE), exploit the arterial route to selectively deliver therapy to tumors. However, these therapies have several limitations, urging the development of new treatment strategies [6, 7].

Ethiodized oil is an FDA-approved radio-opaque agent composed of a variety of oils including the ethyl esters of fatty acids of poppy seed oil. Ethiodized oil can be targeted to hepatic tumors through selective injections via the hepatic artery, where it is able to embolize hepatic tumors, accumulating and remaining in the tumors while clearing out of normal liver tissue [8, 9]. The selective targeting and embolization of hepatic tumors makes ethiodized oil an excellent vehicle for the selective delivery of therapeutic radionuclides. β-particle emitting radiopharmaceutical therapies, such as the commercially available Yttrium-90 glass or resin microspheres have been developed over the past 20 years [6, 10] but have shown limited survival benefits in patients with HCC as compared to TACE, the standard of care treatment option. Compared to Yttrium-90 microspheres that accumulate into arterioles, β-emitting agents, such as [^131^I]I-ethiodized oil, seem to be directly up taken by tumor cells, resulting in a higher tumor radiation dose [11]. However, HCC patients treated with [^131^I]I-ethiodized oil vs. TACE had similar survival up to three years post-treatment; yet patients that had a portal vein thrombosis or more advanced disease demonstrated a significantly higher mean survival as compared to patients treated with TACE [11]. The incorporation of Iodine-131 into ethiodized oil is costly and the exploration of alternate β-particle emitting radionuclides has been explored, such as a Rhenium-188 chelate emulsified into ethiodized oil [12–14]. Despite initial efficacy of the [^188^Re]Re emulsion, the increased hepatic and hematologic toxicities have been a concern [14]. In addition, the low energy transfer of β-particles introduces the ability of the remaining tumors to develop resistance mechanisms further limiting the treatment options for patients [15, 16].

**Fig 1:**
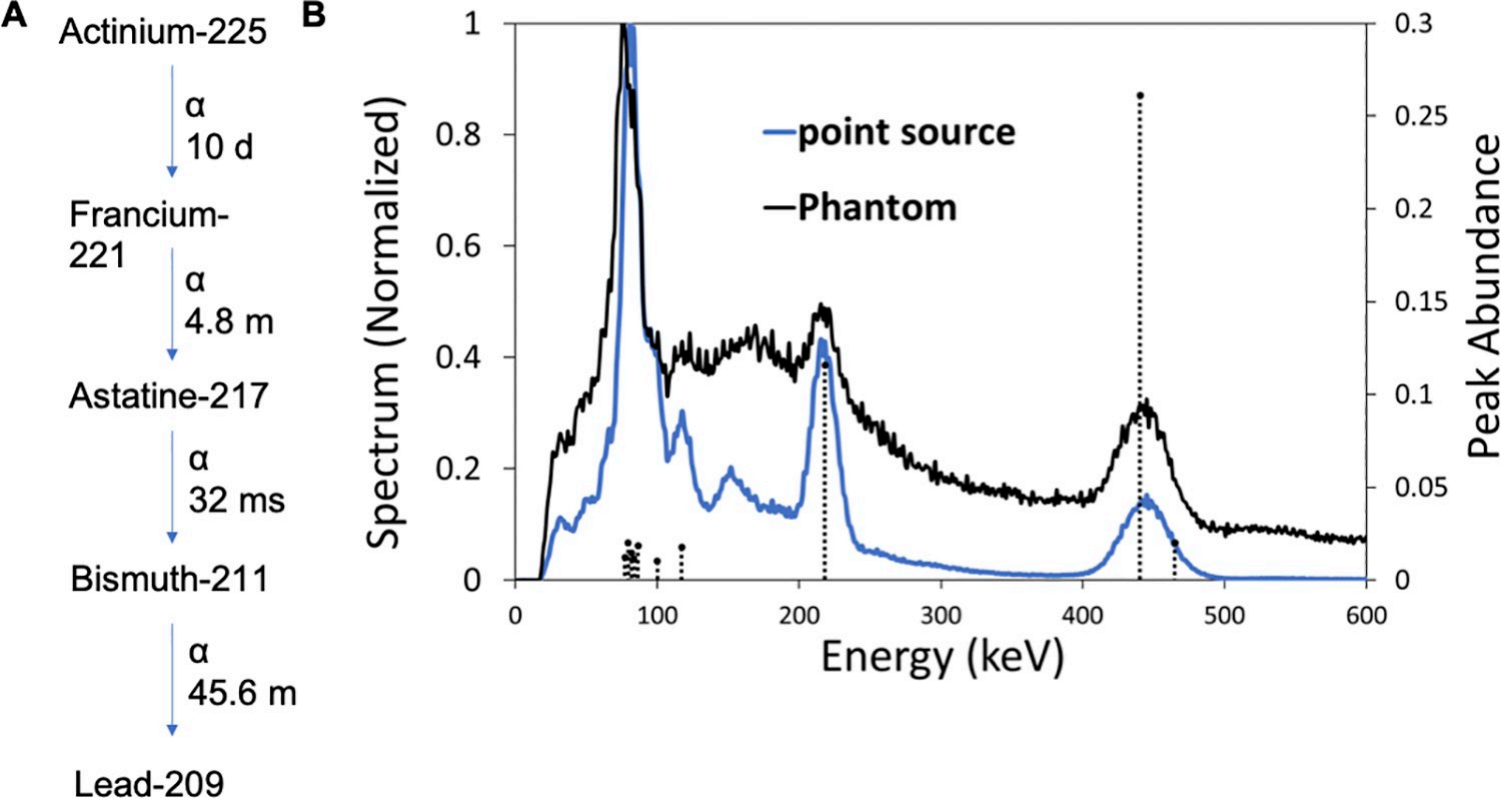
Decay scheme and energy spectrum of Actinium-225. (A) Actinium-225 decay scheme [simplified] (B) Energy spectrum of Actinium-225 from a Siemens’ SPECT system). Dashed lines indicate gamma and X-ray emissions and their abundance, including 218 keV representing Francium-221 and 440 keV representing Bismuth-213 for SPECT imaging.

Targeted alpha therapy (TAT) has emerged as a highly potent treatment for cancer [17–19]. The high potency of TAT impacts normal tissue as well, highlighting a need to accurately determine absorbed doses to provide well-defined dosing strategies and effective treatment while minimizing toxicities. Currently, surrogate imaging agents are utilized to evaluate TAT agents; however, complex decay schemes of α-particle emitting radionuclides are not accurately portrayed using surrogate imaging strategies, urging the development of methodologies and instrumentation to image α-particle emitting radionuclides. Actinium-225 is one of the α-particle emitting radionuclides currently being explored in the clinic. Actinium-225 has a complex decay scheme and energy spectrum (Fig 1). The decay of Actinium-225 and its daughters primarily releases energy as α-particles (Fig 1A); however, γ-emissions suitable for SPECT imaging occur (i.e. at 218 keV and 440.45 keV), but are in low abundance (Fig 1B). Please note, eventhough there are several low abundant X-rays from Actinium-225 and its decay daughters at the 70 keV to 90 keV energy range, the peak around 80 keV shown in Fig 1B is dominated by the lead X-rays generated from interactions of high-energy photons with the collimator. This is a well known phenomina in SPECT physics [20]. In addition, this region is also heavily contaminated by downscatter, and thus should not be used for imaging on current clinical SPECT systems. The activity administered for TAT is extremely small due to their high potency, compounding the low abundance of rays suitable for imaging and resulting in a small number of γ photons for SPECT imaging. Furthermore, the initial α-particle decay with its high recoil energy breaks the bonds within the chelate, resulting in the release of the free decay daughters. These decay daughters may migrate to other organs, complicating dosimetry calculations. Surrogate imaging radionuclides do not replicate the complex decay schemes that are commonly found for α-particle emitting radionuclides. Therefore, it is urgent and essential to develop quantitative SPECT imaging methodologies specifically for these TAT radionuclides to improve the accuracy and precision of the imaging and the resulting knowledge of the distributions and dose deposition of the agent and its daughters.

In this study we investigate the potential of an [^225^Ac]Ac-labeled chelate emulsified in ethiodized oil as a TARE agent to selectively target liver tumors. Furthermore, we evaluated the prospect of using a clinical SPECT/CT system to image and monitor the distribution of the [^225^Ac]Ac-labeled TARE agent as well as its daughters in the VX2-tumor bearing rabbit model over a 6-day window.

## Materials and methods

### Reagents

All chemicals were purchased from Sigma-Aldrich Chemical Co. (ST. Louis, MO, USA) or Thermo Fisher Scientific (Pittsburgh, PA, USA), unless otherwise specified. DOTA-tris(tert-butyl ester) was purchased from Macrocyclics, Inc. (Dallas, TX, USA). Indium-111 ([^111^In]InCl3) was purchased from BWZXT ITG Canada, Inc. (Ottawa, ON, Canada). Actinium-225 nitrate was purchased from Oak Ridge National Laboratory (Oak Ridge, TN, USA).

#### Chemical syntheses

*DOTA-tris(t-Bu ester)-TDA* was synthesized as previously described with modifications [21]. Briefly, the DOTA-tris(t-Bu) ester (50 mg, 0.09 mmol) and HBTU (0.168 mg, 0.44 mmol) were dissolved in 2 mL of DMF. The resulting solution was stirred for 30 minutes at room temperature (RT) then 1-tetradecylamine (TDA) (22 mg, 0.11 mmol) and DIPEA (76 μl, 0.44 mmol) were added to the solution, which was stirred overnight at RT. The solution was extracted with ethyl acetate washed with water (2x) then brine (1x). The ethyl acetate solution was dried over MgSO_4_, filtered and the ethyl acetate was evaporated under vacuum to yield a yellow powder. The DOTA-tris(t-Bu ester)-TDA was purified by silica gel chromatography (eluent: hexane/ethyl acetate, 3/1 v/v), purity confirmed by TLC and the product was used without further confirmation.

*DOTA-TDA*. DOTA-tris(t-Bu ester)-TDA (30 mg, 0.04 mmol) was dissolved in trifluoroacetic acid (125 μl) and dichloromethane (400 μl) and stirred overnight at RT. The solvent was evaporated under vacuum yield a pure white solid, 51.3% was recovered. DOTA-TDA was successfully synthesized (overall yield 53.1%) and confirmed by mass spectrometry. DOTA-TDA was confirmed by using a Thermo Scientific Q Exative Plus Orbitrap Mass Spectrometer coupled with a Vanquish UPLC system at the Metabolomics Facility at Johns Hopkins School of Medicine (Director–Dr. Anne Le) (M+H): calculated 600.4336, found 600.4316; (M-H) calculated 598.4185 found 598.4203.

#### Radiolabeling of DOTA-TDA

For radiolabeling the following were added to acid wash Eppendorf tube 1 μl of Indium-111, 40 μl of 0.4M NaOAc buffer pH = 4.5, and 1 μl of DOTA-TDA (1 μg/μl). The solution was heated at 95°C for 15 minutes and purified using a Waters Sep-Pak C18 (Milford, MA, USA). [^111^In]In-DOTA-TDA was eluted with ethanol, dried, and resuspended in sterile PBS with greater that 95% radiopurity as determined by thin layer chromatography (TLC).

For Actinium-225, 5 μl of Actinium-225 solution, 5 μl of 2M Tris buffer pH = 7, and 2.5 μL of DOTA-TDA were added to an acid washed tube, heated at 95°C for 15 minutes. Radiolabeling yields were ≥95%; [^225^Ac]Ac-DOTA-TDA was used without further purification.

#### Partition coefficient (Log P)

Log P were determined for the radiolabeled conjugates, [^111^In]In- or [^225^Ac]Ac-DOTA-TDA, were dissolved in 1 mL of octanol in a centrifuge tube. The centrifuge tube was vigorously vortexed for a minute with 1 mL of 0.9% saline. The tube was centrifuged at 1000 rpm for 5 minutes and allowed to sit for 30 minutes until the phases were clearly separated. The organic phase (Octanol) and the aqueous phase (Saline) were collected separately and counted at equilibrium of Actinium-225 in an automatic γ-well counter (Perkin-Elmer 2470 WIZARD^2^® Automatic Gamma Counter, Waltham, MA, USA). The Log P values were calculated using the following formula: Log P = Log ([Counts]_octanol_/[Counts]_saline_).

### Animal studies

This study was carried out in strict accordance with the recommendations in the Guide for the Care and Use of Laboratory Animals of the National Institutes of Health. The protocol was approved by the Animal Care and Use Committee of the Johns Hopkins University School of Medicine (Protocol Number: M019M321; RB19M81). All the animals in this study were monitored daily for health and behavior. Rabbit surgery was performed under a mixture of ketamine and xylazine, and the anesthesia was maintained with isoflurane in oxygen. The rabbits were euthanized at pre-determined timepoints using Ethansol®. The mice were euthanized at pre-determined timepoints by isoflurane overdose and cervical dislocation. Mice in the survival study were observed for signs of pain and distress (lethargy, hunched back, paralysis, etc.), weight loss, and tumor size [volume = 0.5 x (length x width^2^)]. The mice in the survival study were euthanized as described above if one of the following conditions were met: 1. weight loss (>20%); 2. primary tumor reaching 1,500 mm^3^; 3. signs of pain or distress; or 4. 180 days post-treatment. In this study a total of 33 NCG male mice and 3 adult New Zealand rabbits were used. All efforts possible were made to minimize suffering of the animals in this study. All sacrificed animals met the described end-point criteria, no animals died prior to end-points.

Animal studies were performed using 7-8-week-old healthy male NCG mice obtained from Charles River Laboratories (Wilmington, MA, USA). Adult New Zealand rabbits were used weighing approximately 4 kg (Myrtle’s Rabbitry, Thompson Station, TN, USA). For tumor retention and survival studies the mice were injected subcutaneously in the right flank with approximately 1 x 10^6^ HEP2G cells in 100 μL of sterile PBS and Matrigel (1:1).

#### Mouse model

*Tumor retention*. Biodistribution studies were carried out as previously described in healthy NCG male mice (n = 3/group) bearing HEP2G subcutaneous tumors [22, 23]. To assess tumor retention the mice were divided into two groups that were injected intratumorally with [^111^In]In-DOTA-TDA (37 kBq, 60 μL) alone or [^111^In]In-DOTA-TDA (37 kBq, 60 μL) emulsified in ethiodized oil. At 48-hours post-injection (p.i.), the mice were euthanized. The blood, heart, lungs, liver, spleen, kidneys, stomach (with content), intestines (with content), bone, muscle, and tumors were harvested, weighed, and measured in an automatic γ-well counter. The percentage of injected dose per gram (%ID/g) was calculated by comparison with a weighed, diluted standard.

*Therapeutic efficacy*: *Survival studies*. Subcutaneous liver cancer models were used to evaluate the preliminary therapeutic efficacy of [^225^Ac]Ac-DOTA-TDA/ethiodized oil emulsions. The following treatments were administered in subcutaneously HEP2G tumors (266.5 ± 23.2 mm^3^): 1. Saline (60 μL) 2. Ethiodized oil alone (60 μL) or 3. [^225^Ac]Ac-DOTA-TDA/ethiodized oil (37 kBq, 60 μL). Survival fractions were plotted as a Kaplan-Meier survival curve using Prism 8 (GraphPad; La Jolla, CA, USA).

#### VX2 rabbit model

*Anesthesia*. Rabbits were administered a mixture of ketamine (30–50 mg/kg) and xylazine (3–5 mg/kg) intramuscularly prior to tumor harvesting, implantation, and imaging. Anesthesia was maintained with 3% isoflurane in oxygen as well as additional injections of the ketamine/xylazine solution.

*Ultrasound guided tumor implantation*. The implantation of the VX2 tumor was done as previously described with modifications [24]. Briefly, the solid VX2 tumor for implantation was obtained from a carrier rabbit that had been injected intramuscularly in the thigh approximately 2 weeks prior. The VX2 tumor was harvested, placed in 0.9% sodium chloride, and minced into small pieces. The minced tumor was prepared in a 16-gauge Angiocath (2 inches long), using ultrasound guidance, the needle punctured the liver and the tumor pieces were pushed into the liver with a guide wire. Manual compression was administered after the needle was removed.

*Intrahepatic arterial injection including agents and dosing*. Hepatic angiography was performed as previously described [24]. Briefly, rabbits with VX2 hepatic tumors, confirmed by ultrasound were anesthetized and surgical cutdowns were performed to expose the femoral artery. A 4-F sheath (Cook) was inserted to allow a 2-F catheter tip (JB1 catheter; Cook) to access the common hepatic artery. Fluoroscopy was utilized to select the arterial branch and radioembolization was performed.

*SPECT imaging in VX2-bearing rabbit*. SPECT/CT imaging was performed on a Siemens Symbia T16 SPECT/CT system. The anesthetized rabbit (n = 1) was placed supine on the imaging bed with the whole body in the field-of-view. The images were acquired at 24 hours, 72 hours and 6 days p.i. Projection data were acquired in 120 views over 360° with a 128 x 128 matrix and 4.795 mm pixel size. The imaging time were 75 seconds per view for the 24-hour imaging, and 90 seconds per view for the 72-hour and 6-day imaging. The high-energy collimators were used. Data were binned into two energy windows—20% centered at 440.45 keV peak of Bismuth-213 and 20% centered at 218.2 keV peak of Francium-221, respectively. Both isotopes are daughters of Actinium-225 decay and emit gamma photons that can be imaged by SPECT. Francium-221 has a short half-life of 4.9 minutes, therefore its distribution represents those of Actiniun-225’s. Bismuth-213 has a half-life of 45.6 minutes and could have redistributed from the parent isotope. After SPECT acquisition, a CT scan was performed to provide anatomical images and an attenuation map. The SPECT images were reconstructed with Siemens Flash3D OSEM algorithm with compensation for attenuation and resolution [25]. A total of 6 iterations with 15 subsets per iteration were used.

*SPECT imaging analysis*. Volumes of interest (VOIs) were drawn on the SPECT/CT images for the normal organs/tissues liver, kidneys, muscle (left thigh muscle) and the tumor using the software VivoQuant^TM^ version 4.0 patch 3 (Invicro Imaging Services and Software, Boston, MA, USA) using a method developed by Plyku et al. [26]. The method was developed for tumor countering for SPECT imaging to take into consideration the spill-out or partial volume effect, the method has also been used for small normal tissues/organs as the pituitary gland [27].

Ex vivo *biodistribution VX2-bearing and non-tumor bearing rabbits*. Biodistribution studies were carried out following the SPECT imaging approximately 6-days post-injection of [^225^Ac]Ac-DOTA-TDA (0.49 MBq) emulsified in 1 mL of ethiodized oil in the VX2-bearing rabbit (n = 1) and at approximately 24 hours for non-tumor bearing rabbits (n = 2). At the end timepoint the rabbits were euthanized (Ethansol®, 1 mL minimum then 1mL/4.5 kg). The blood, heart, lungs, liver, spleen, kidneys, stomach, intestines, marrow, femur+marrow, muscle, gall bladder, bile, and if applicable the tumor were harvested, weighted, and measure in an automatic γ-well counter and/or a Capintec CRC-7 dose calibrator at equilibrium of Actinium-225. The percentage of injected dose per gram (%ID/g) was calculated based on the dose injected and converted to Bq/g using an efficiency coefficient.

**Statistical Analysis** was performed using the software Graphpad Prism 8. All data are presented as mean ± SD. Tumor retentions were compared using 2-way ANOVA. Survival studies used Kaplan-Meier curves that were analyzed using the Log Rank (Mantel-Cox) test. Values were considered significant when *P*<0.05.

## Results

### Radiochemistry

[^111^In]In-DOTA-TDA was radiolabeled at a specific activity of 2.13 MBq/nmol with greater than 95% radiopurity following purification. The [^225^Ac]Ac-DOTA-TDA was radiolabeled at a specific activity of 0.36 MBq/nmol with greater than 95% radiolabeled yield.

### Determining partition coefficients (LogP)

The LogP for [^111^In]In-DOTA-TDA was 1.57 ± 0.01 and the LogP for [^225^Ac]Ac-DOTA-TDA was 1.65 ± 0.02. For comparison, doxorubicin, a chemotherapeutic used for TACE, has a reported LogP of 1.27 [28].

### Tumor retention of ^111^In-DOTA-TDA emulsion

**Fig 2:**
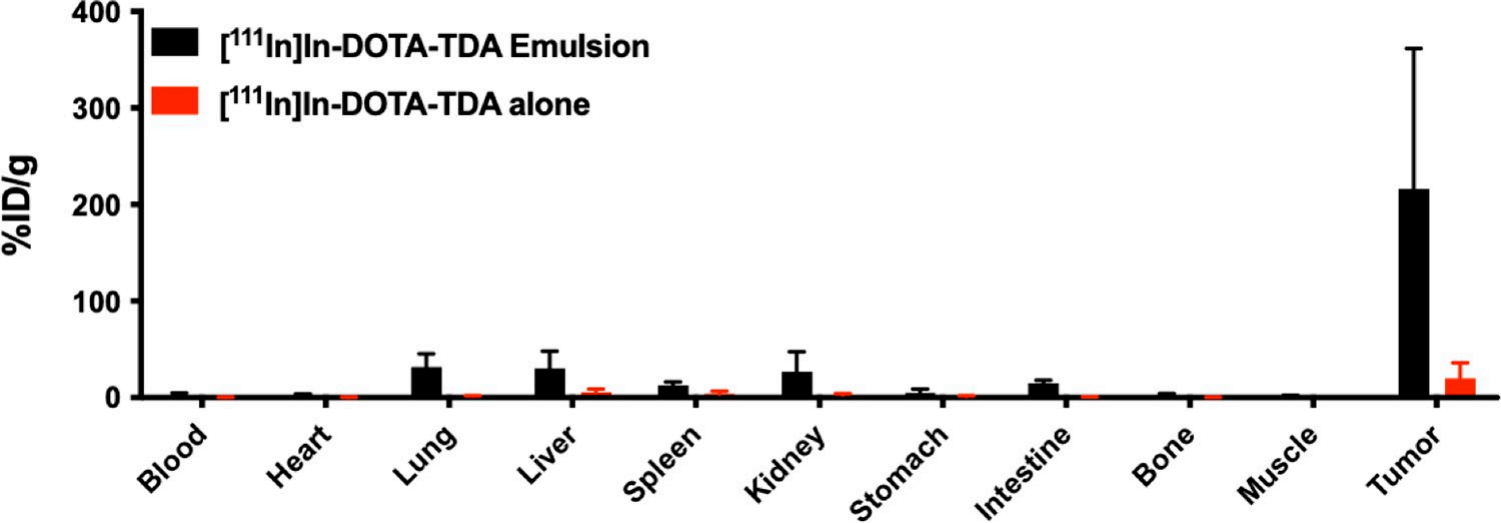
Biodistribution data in tumor-bearing mice. Biodistribution of [^111^In]In-DOTA-TDA (37 kBq) with and without ethiodized oil emulsification in Hep2G tumor-bearing NCG mice at 48 hours post-injection (n = 3, p≤0.0001).

The retention of [^111^In]In-DOTA-TDA alone or as an emulsion (Fig 2, S1 Table) were compared 48-hours p.i. using a 2-way ANOVA test in HEP2G tumor-bearing mice. The [^111^In]In-DOTA-TDA emulsion had significantly higher uptake within the tumor as compared to [^111^In]In-DOTA-TDA alone after 48 hours (216±145%ID/g vs. 20.0±15.9%ID/g; p≤0.0001), supporting the statement that ethiodized oil is able to retain the proposed agents in a model of liver cancer.

### Therapeutic efficacy: Survival studies

**Fig 3:**
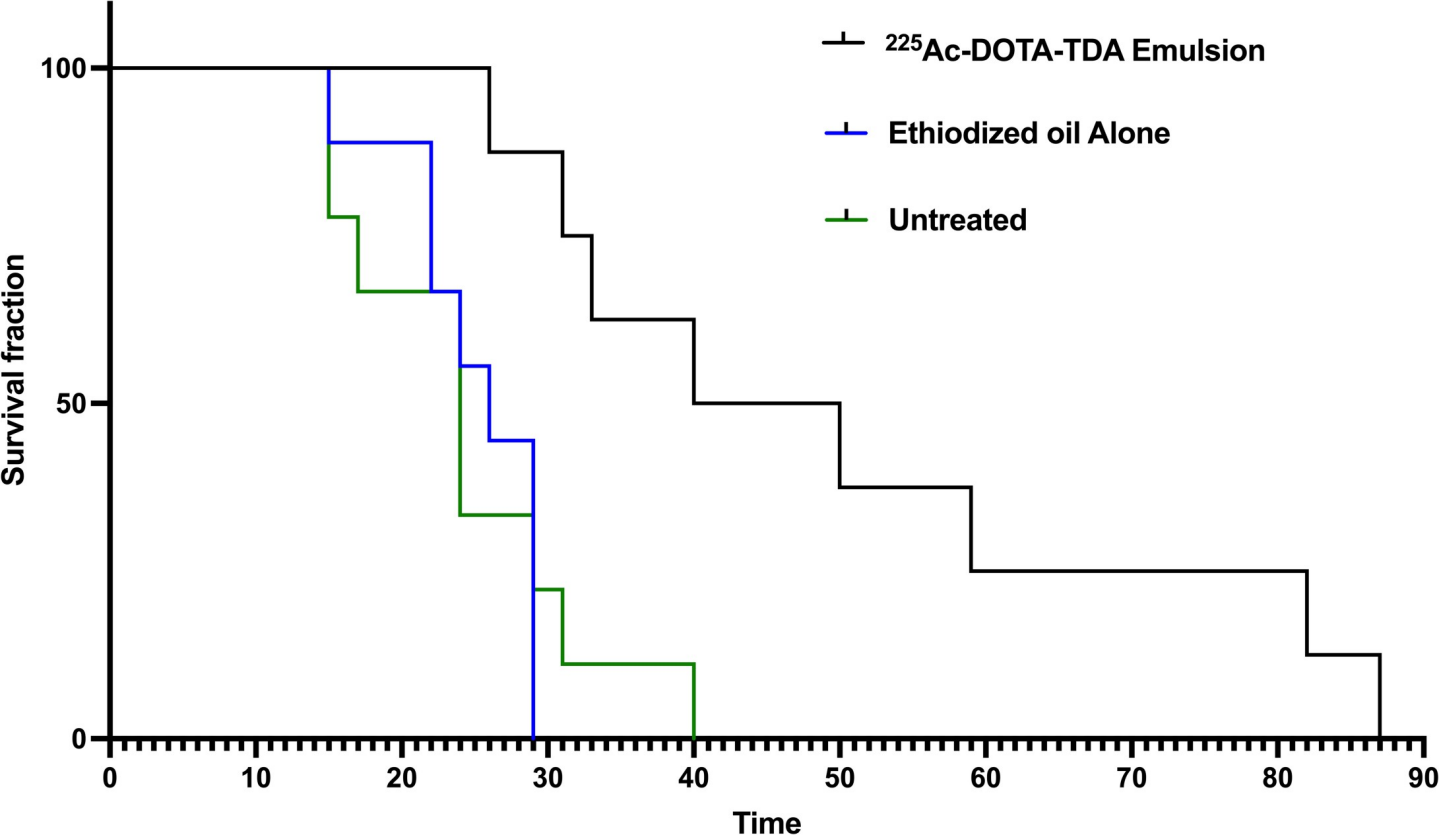
Survival plots of tumor-bearing mice. Kaplan-Meier survival plots in Hep2G-tumor bearing mice receiving the following treatments: 1. Saline (60 μL) 2. ethiodized oil alone (60 μL) or 3. ^225^Ac-DOTA-TDA emulsion (37 kBq, 60 μL).

**Fig 4:**
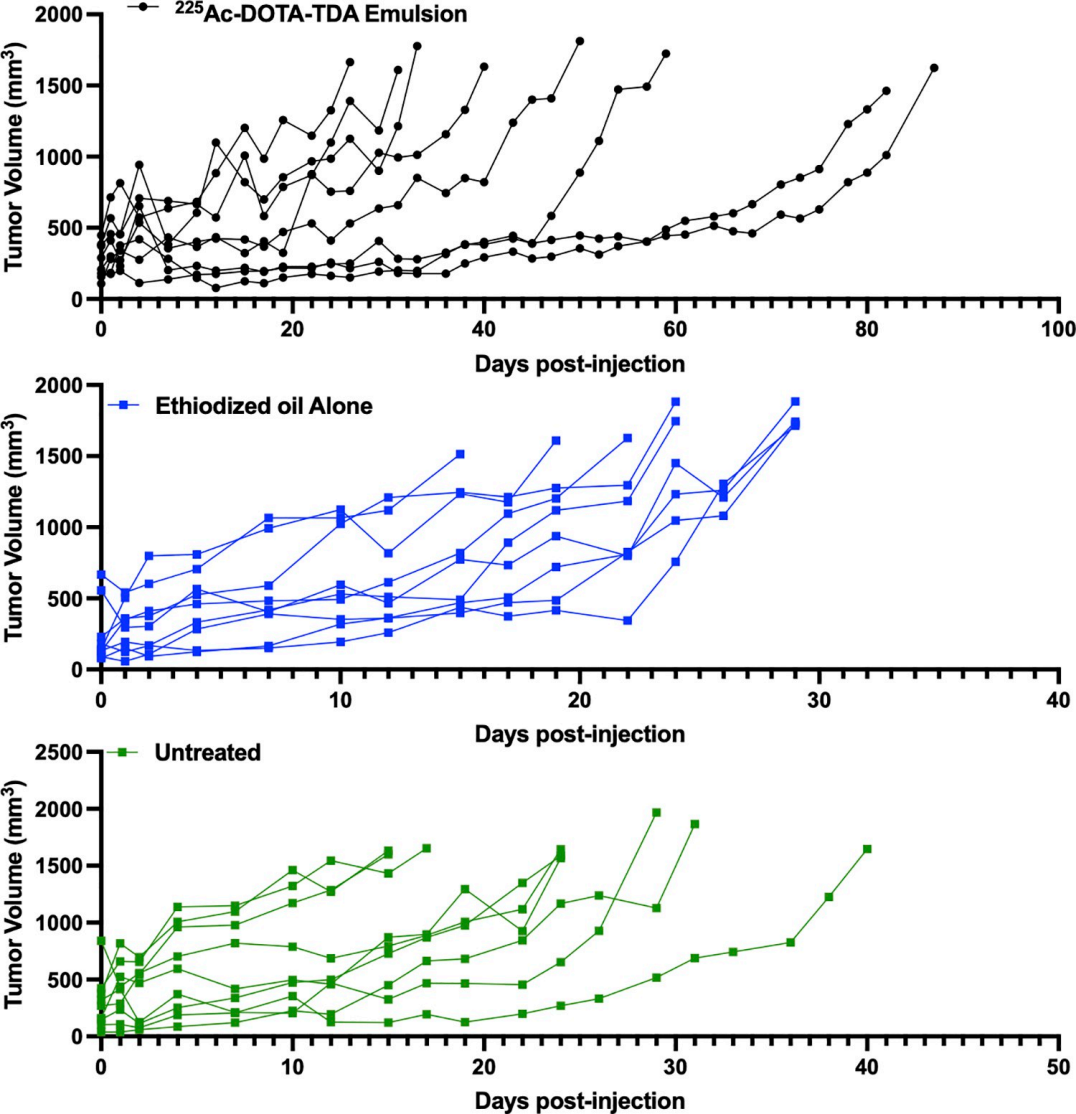
Individual tumor growth curves in a subcutaneous model of liver cancer. NCG mice were injected in the right flank with HEP2G cells when the tumors reached an average volume of 266.5 ± 23.2 mm^3^ the following treatments were administered intratumorally: 1. Saline (60 μL) 2. ethiodized oil alone (60 μL) or 3. [^225^Ac]Ac-DOTA-TDA emulsion (37 kBq, 60 μL).

Kaplan-Meier survival curves (Fig 3, S2 Table) showed significant improvement in survival of HEP2G tumor-bearing mice when treated with [^225^Ac]Ac-DOTA-TDA emulsions as compared to both the untreated control (p<0.005) and ethiodized oil alone (p<0.001). The median survival for [^225^Ac]Ac-DOTA-TDA emulsions in treated mice was 45 days, ranging from 26–87 days, as compared to 24-days (untreated, [15–29 days]) and 26-days (ethiodized alone, [15–40 days]). In addition, the rate of tumor growth in the [^225^Ac]Ac-DOTA-TDA emulsions treated group was slowed as demonstrated by the individual tumor growth curves (Fig 4, S3–S5 Tables).

### SPECT imaging in VX2-bearing rabbit

**Fig 5:**
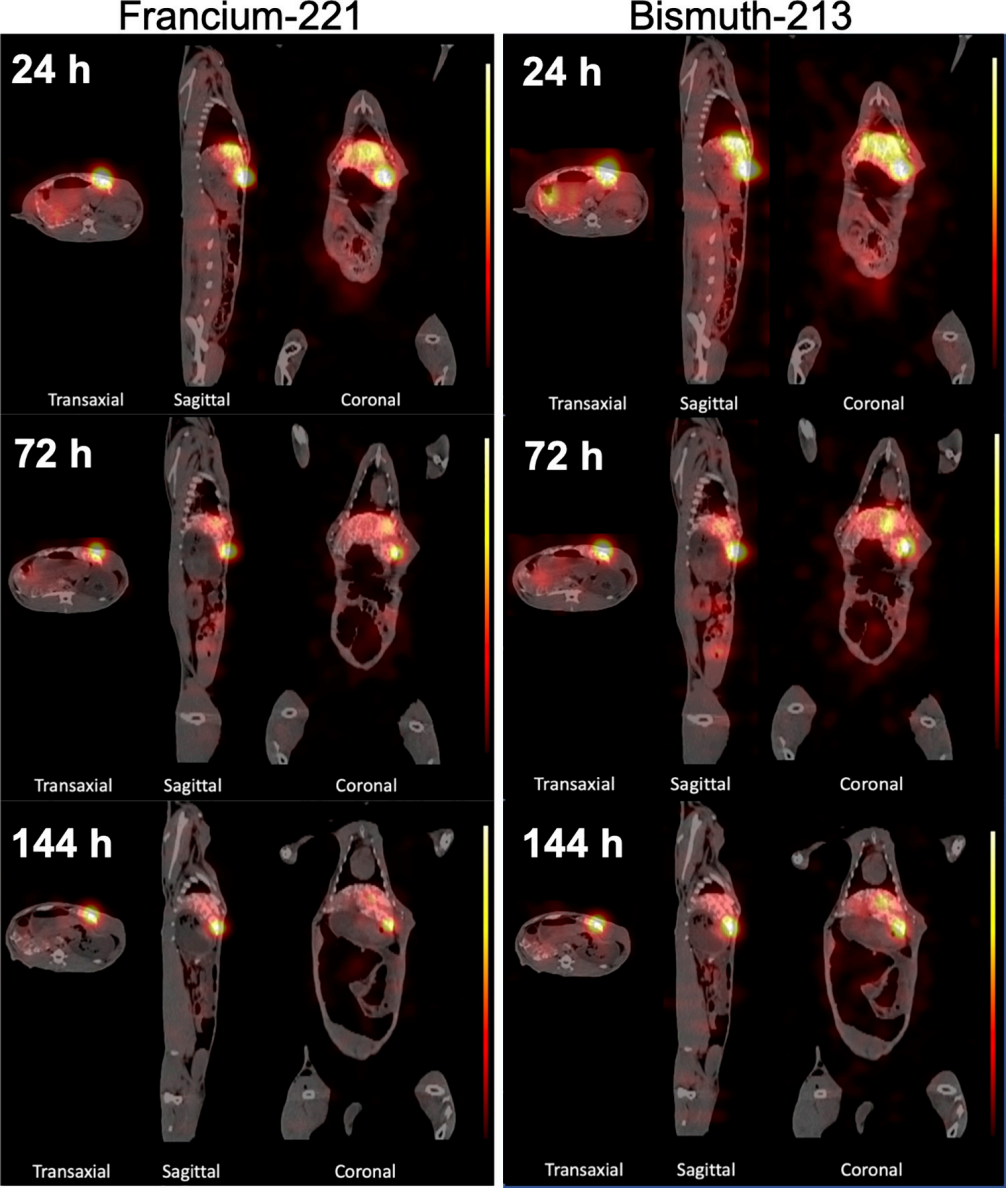
SPECT/CT images of a rabbit with VX2 hepatic tumor. Fused SPECT/CT images of rabbit with a VX2 hepatic tumor at 24, 72 and 144-hours post-injection of [^225^Ac]Ac-DOTA-TDA emulsion. Left Panels: Francium-221 energy window. Right panels: Bismuth- 213 energy window.

The fused SPECT/CT images from 24, 72, and 144-hours p.i. (Fig 5) demonstrated that the activity of [^225^Ac]Ac-DOTA-TDA emulsion agent accumulated mainly in the VX2 tumor with gradual clearance out of normal liver tissue. At 72 hours and 6-day p.i., the [^225^Ac]Ac-DOTA-TDA emulsion agent is concentrated within the tumor; however, the figures clearly show that there were differences in the distributions between the Francium-221 energy window and Bismuth-213 energy window—there was increased signal in the normal liver tissue above the tumor in the Bismuth-213 window, most likely associated with the escape of the free Bismuth-213 daughter from TARE agent.

### SPECT imaging analysis

The tumor to liver ratio on the SPECT/CT images regarding the Francium-221 energy window for 24, 72 hours and 6-days were approximately 23:1, 20:1 and 27:1, respectively, and the corresponding tumor to muscle ratios were 265:1, 207:1 and 191:1, respectively. Regarding the Bismuth-213 energy window the tumor to liver ratio on the SPECT/CT images for 24, 72 hours and 6-days were 15:1, 16:1 and 21:1, respectively, and the corresponding tumor to muscle ratios were approximately 186:1, 141:1and 118:1, respectively.

### *Ex-Vivo* biodistribution in VX2-bearing rabbit

Following the SPECT imaging, biodistribution studies were carried out in the VX2-tumor bearing rabbit at 6-days p.i. of the [^225^Ac]Ac-DOTA-TDA emulsion (**Table 1**). Accumulation of the TARE agent was predominately in the VX2 tumor (40.6%ID/g). The next highest uptake was seen in the normal liver tissue (0.23%ID/g), resulting in having approximately 177:1 tumor to liver ratio (**Table 2**). The TARE agent accumulation in normal tissues were minimal, only slightly above background as determined by uptake in the muscle (0.001%ID/g). This low uptake in normal tissue is consistent with the *ex-vivo* biodistributions in non-tumor bearing rabbits (n = 2) at approximately 24-hours p.i. via the hepatic artery as shown in **Table 1**.

**Table 1:** *Ex vivo* biodistribution of [^225^Ac]Ac-DOTA-TDA in a technical rabbit model.

	Tumor-bearing 6 days p.i.*		Non-tumor bearing 23.5 hours p.i.*		Non-Tumor bearing 28 hours p.i.*	
	%ID/g	Bq/g	%ID/g	Bq/g	%ID/g	Bq/g
**Blood**	**0.001**	**4.291**	**0.006**	**161.4**	**0.036**	**592.2**
**Heart**	**0.002**	**5.641**	**0.003**	**94.95**	**0.004**	**59.36**
**Lungs**	**0.014**	**46.23**	**0.028**	**781.0**	**0.023**	**382.0**
**Liver**	**0.233**	**753.2**	**0.153**	**4352**	**0.172**	**2796**
**Spleen**	**0.092**	**296.4**	**0.078**	**2214**	**0.092**	**1495**
**Kidneys**	**0.020**	**64.59**	**0.039**	**1114**	**0.045**	**731.0**
**Stomach**	**0.010**	**30.65**	**0.047**	**1346**	**0.044**	**708.8**
**Intestines**	**0.020**	**826.3**	**0.031**	**880.3**	**0.028**	**449.9**
**Marrow**	**0.018**	**66.02**	**0.013**	**372.1**	**0.013**	**210.1**
**Whole Bone**	**Indistinguishable from background**		**0.054**	**1521**	**0.011**	**182.7**
**Muscle**	**0.001**	**2.637**	**0.001**	**18.54**	**0.002**	**26.93**
**Gall Bladder**	**0.081**	**260.134**	**0.098**	**1600.62**	**0.105**	**2986.55**
**Bile**	**0.023**	**74.533**	**0.091**	**1483.13**	**0.064**	**1812.35**
**Tumor**	**40.62**	**148370**	**---**		**---**	

*Organs were counted at equilibrium of Actinium-225 and its daughters.

**Table 2:** Tumor ratios based on SPECT imaging of francium-221 and bismuth-213.

	Francium-221		Bismuth-213	
Time p.i	Tumor:Liver	Tumor:Muscle	Tumor:Liver	Tumor:Muscle
**24 h**	23:1	265:1	15:1	186:1
**72 h**	20:1	207:1	16:1	141:1
**6 d**	27:1	191:1	21:1	118:1

## Discussion

Due to its indolent course, most patients with HCC have advanced and unresectable disease at the time of diagnosis and are being offered only non-surgical palliative treatment options [5, 6]. In addition, the liver is also the most common site for metastatic disease, including colorectal liver metastases. Intra-arterial therapies are widely used for treatment of patients with HCC or metastatic liver cancer [5]. Nevertheless, these therapies have shown limited survival benefit for patients with HCC, urging the need for developing novel improved therapeutic strategies [5, 29–33].

β-emitting radiopharmaceutical agents have shown promising results but the survival benefits in patients with HCC are comparable to the standard of care, TACE [34]. TAT provides highly potent agents [35–48] that are highly damaging to tumor cells, effectively causing irreparable DNA damage to tumor cells, needing only a few α-tracks as compared to 10^3^−10^4^ tracks for a β-emitting agents. Consequently, there is growing interest in TAT agents for cancer therapy [49–51]. Recently, the first in human experience with [^213^Bi]Bi-DOTATOC, a TAT agent for neuroendocrine cancer, was selectively targeted via the hepatic artery to treat metastatic disease in the liver of neuroendocrine cancer patients not responding to standard therapies as well as [^177^Lu]Lu-DOTATOC, a β-emitting targeted therapy. Kratochwil et al. demonstrated in a small subset of patients that [^213^Bi]Bi-DOTATOC was able to overcome refractory disease and provided positive responses in patients [52]. Biomarkers for targeted radiopharmaceutical therapies are limited for HCC; however, the selective deliver of therapies via the hepatic artery for primary liver cancers are common. In this study, we investigated the potential to exploit intra-arterial delivery of the TARE agent, [^225^Ac]Ac-DOTA-TDA emulsion, for its ability to target primary liver tumors as well as its therapeutic efficacy in a mouse model of HCC.

Ethiodized oil has been demonstrated to have high selectivity and retention in the liver [53]; Ethiodized oil has shown to be retained in hepatic tumors for months [54]. [^188^Re]Re-labeled chelators, modified to introduce lipophilicity, have been successfully incorporated into ethiodized oil for β-emitting radiopharmaceutical therapy [55]. Here, we used a similar approach and altered the DOTA chelator, which is suitable for [^225^Ac]Ac-labeling, by incorporating an alkyl side chain to introduce lipophilicity. The resulting agent, DOTA-TDA, was radiolabeled with both Indium-111 and Actnium-225 in high radiolabeling yields and purities. Both the [^111^In]In- and [^225^Ac]Ac-DOTA-TDA were shown to be lipophilic as determined by the partition coefficients, helping them to be emulsified. The emulsification of [^111^In]In-DOTA-TDA with ethiodized oil resulted in an approximate 11-fold increase in retention of the radiolabel agent within the mouse model of primary liver cancer. The preliminary potency of the TARE agent, [^225^Ac]Ac-DOTA-TDA emulsions was evaluated and demonstrated significant increases in survival in treated mice as compared to mice receiving saline or ethiodized oil alone. [^225^Ac]Ac-DOTA-TDA emuslsions treated mice almost doubled (1.8x) their median survival as compared to the control groups, with the first treated mouse not reaching its endpoint until the median survival of the control. The preliminary results in the mouse model of primary liver cancer are promising, and we further evaluated the alpha-emitting TARE agent in a technical model to confirm selective delivery and retention via intra-arterial delivery.

The rabbit VX2 model provides a technical animal model for intra-arterial injections, allowing us to explore the selective delivery of [^225^Ac]Ac-DOTA-TDA emulsions to a hepatic tumor. We have confirmed delivery, accumulation, and retention of the TARE agent within the VX2 tumor by *ex-vivo* biodistribution, and *in-vivo* SPECT imaging of Acintium-225’s daughters (Francium-221 and Bismuth-213) using a clinical SPECT/CT system. Even though feasibility of SPECT imaging of Actinium-225 decay chain has been demonstrated [56, 57]—to our knowledge, this work is the first to demonstrate it can be done longitudinally *in-vivo* on a clinical SPECT/CT system with low activities. Furthermore, the fact that the two energy windows showed different tumor to liver/muscle ratios indicates the importance of using SPECT to monitor both parent and daughter isotopes. Clearance from normal tissues including the liver was seen in early biodistribution studies in non-tumor-bearing rabbits at approximately 24 hours p.i. of [^225^Ac]Ac-DOTA-TDA emulsions with the highest uptake, less than 0.2%ID/g, in the normal liver. The VX2-bearing rabbit’s biodistribution showed that the normal liver had slightly higher uptake (0.23%ID/g) of [^225^Ac]Ac-DOTA-TDA emulsion at 6-days p.i., most likely associated with clearance of the TARE agent from the VX2 tumor, which had 40.6%ID/g uptake at 6-days p.i. This is further confirmed by the SPECT images that demonstrated the stark contrast of the TARE agent within the tumor as compared to the normal liver tissue as well as the increase in the tumor to liver ratio at 6-days p.i. More importantly, the SPECT images also demonstrated the differences in the distribution of Actinium-225/Francium-221 from that of free Bismuth-213, indicating the importance of SPECT imaging for Actinium-225-labeled agents.

## Conclusion

In summary, these studies demonstrate that the developed TARE agent, [^225^Ac]Ac-DOTA-TDA emulsions has promise as a therapeutic agent for hepatic tumors, including primary liver tumors. This work highlights that the combination of chelator labeled with a long-lived α-emitting radionuclide emulsified with ethiodized oil is capable of being selectively delivered to hepatic tumors, delivering a highly potent therapeutic dose to hepatic tumors over an extended period of time. Furthermore, we have shown that the [^225^Ac]Ac-labeled TARE agent can be imaged and longitudinally monitored through clinical SPECT imaging using its daughters Francium-221 and Bismuth-213 even with very low injected activity. The development of SPECT imaging methodologies to monitor the complex decay scheme of α-emitting radionuclides, such as Actinium-225, and their daughters are important for accurately monitoring the distribution of TAT agents *in-vivo* over time through molecular imaging providing essential pharmacokinetic data to optimize the therapeutic efficacy. More importantly, these SPECT imaging methodologies can be implemented for [^225^Ac]Ac-labeled TAT agents currently in clinical trials helping to develop well-defined dosing strategies and optimal patient-specific treatment planning.

## Supporting information

S1 Table Raw %ID/g for biodistribution of [^111^In]In-DOTA-TDA emulsion and free. (PDF)

S2 Table Raw data for Kaplan-Meier survival plots in Hep2G-tumor bearing mice. (PDF)

S3 Table Volume of HEPG2 tumors in mice treated with ^225^Ac-DOTA-TDA emulsion. (PDF)

S4 Table Volume of HEPG2 tumors in mice treated with ethiodized oil alone. (PDF)

S5 Table Volume of HEPG2 tumors in mice treated with Saline. (PDF)
