# Supplementary material for: Preliminary evaluation of alpha-emitting radioembolization in animal models of hepatocellular carcinoma
Source: PLoS One. 2022 Jan 21;17(1):e0261982. doi: 10.1371/journal.pone.0261982 (PMC8782514; doi:10.1371/journal.pone.0261982)
Supplement: S1 Table — (PDF) [file pone.0261982.s001.pdf]

	48 Hours w/ 111In-DOTA-TDA Emulsion			48 Hours 111In-DOTA-DOTA Alone		
Blood	0.95	5.08	2.8	0.22	0.32	0.37
Heart	0.95	4	2.44	0.13	0.32	0.44
Lung	41.91	37.1	15.97	1.05	1.27	1.89
Liver	12.63	48.61	29.1	1.84	6.97	8.06
Spleen	12.22	16.56	9.29	0.94	6.82	3.34
Kidney	8.82	48.88	22.89	1.7	2.67	4.39
Stomach	6.54	7.56	0.4	1.37	0.21	1.85
Intestine	11.59	17.3	16.13	0.7	0.88	0.43
Bone	1.65	4.36	2.2	0.28	0.57	0.61
Muscle	1.16	2.31	1.34	0.16	0.23	0.2
Tumor	65.19	227.5	355.51	37.67	15.09	7.11
