# Supplementary material for: Preliminary evaluation of alpha-emitting radioembolization in animal models of hepatocellular carcinoma
Source: PLoS One. 2022 Jan 21;17(1):e0261982. doi: 10.1371/journal.pone.0261982 (PMC8782514; doi:10.1371/journal.pone.0261982)
Supplement: S2 Table — (PDF) [file pone.0261982.s002.pdf]

Time of death (d)	[^{225}Ac]Ac-DOTA-TDA Emulsion	Ethiodized Oil Alone	Untreated
82	1		
59	1		
50	1		
87	1		
31	1		
26	1		
40	1		
33	1		
29		1	
24		1	
22		1	
29		1	
15		1	
26		1	
29		1	
29		1	
22		1	
31			1
24			1
40			1
15			1
15			1
17			1
29			1
24			1
24			1
