# Supplementary material for: Preliminary evaluation of alpha-emitting radioembolization in animal models of hepatocellular carcinoma
Source: PLoS One. 2022 Jan 21;17(1):e0261982. doi: 10.1371/journal.pone.0261982 (PMC8782514; doi:10.1371/journal.pone.0261982)
Supplement: S3 Table — (PDF) [file pone.0261982.s003.pdf]

Days post-injection	^{225}Ac-DOTA-TDA Emulsion						
0	179.2	206.67	289.132	155.952	373.248	446.7375	107.909
1	176.256	298.5255	410.504	183.2985	456.3365	713.9	281.6
2	196.992	230.917	272.1155	376.648	453.9445	813.6	339.575
4	112.64	652.616	535.5525	419.71	707.5375	573.248	275.562
7	137.214	202.176	355.743	281.775	689.238	638.144	431.694
10	171.396	231.8115	402.1785	148.137	665.334	682.344	366.102
12	176.4	199.962	423.801	77.1375	572.45	884.174	433.35
15	193.55	218.489	417.774	124.6375	1008.0625	1203.052	322.752
17	196	190.125	367.84	110.902	583.2	986.4	405
19	217.328	226.6945	470.016	150.4	787.176	1257.523	324.761
22	215.6	228.152	530	176	870.714	1146.752	878.156
24	256.608	246.5195	410.8275	161.728	1098.5	1326.65	754.443
26	216.6255	249.318	530.45	150.903	1391.6	1663.893	759.755
29	261.121	406.78	635.6335	192.892	1184.625		1026.99
31	182.7525	283.2795	659.45	200.9205	1608.75		993.6
33	177.531	278.5185	851.5625	198.025			1012.11
36	178.292	326.25	744.2	315			1157.65
38	249.9	380.701	849.6	383.496			1328.74
40	292.966	400.078	821.338	382.91			1632.62
43	332.12	444.675	1239.3	424.83			
45	284.2	388.8	1400.7725	390.1365			
47	298.08	584.284	1409.256	414.9925			
50	355.914	887.5125	1812.6795	445.904			
52	311.7465	1110.12		423.612			
54	371.3395	1472.7285		439.078			
57	403.44	1492.1875		402.6275			
59	487.872	1724.976		444.528			
61	548.352			453.125			
64	578.6535			514.83			
66	602.7615			475.136			
68	665.856			460.404			
71	804.816			592.3125			
73	853.05			566.048			
75	912.525			628.452			
78	1229.3855			820.192			
