# Supplementary material for: Preliminary evaluation of alpha-emitting radioembolization in animal models of hepatocellular carcinoma
Source: PLoS One. 2022 Jan 21;17(1):e0261982. doi: 10.1371/journal.pone.0261982 (PMC8782514; doi:10.1371/journal.pone.0261982)
Supplement: S4 Table — (PDF) [file pone.0261982.s004.pdf]

Days post-injection	Ethidodized Oil Alone							
0	178.5215	229.3655	126.976	89.2375	667.8625	133.209	555.2765	78.75
1	124.928	359.5275	350.2845	57.6	540	194.0625	296.225	150.28
2	167.8635	375.584	410	107.3875	601.7375	169.884	303.75	92.5965
4	134.096	525	460	284.746	705.6	332.682	565	124.2
7	150.28	588.7995	482.7095	390.4735	1066.464	419.6625	406.272	165.888
10	194.398	1024.1915	493.3185	351.975	1065.8	530.604	595.35	319.39
12	259.0015	1208.35	612	361	1119.744	510	465.7455	361.978
15	436.982	1245.816	818.928	397.1	1513.8	490	773.6625	469.646
17	374.544	1211.9625	1095.2	470.596		893.101	734.2175	506.626
19	416.5425	1276.224	1202.688	485		1119.008	937.936	721.278
22	344.25	1295.061	1627.208	825.507		1183.952	799.254	811.2
24	758.063	1882.976		1047.8		1745.075	1451.52	1232.01
26	1305			1081.665			1208.35	1260.12
29	1714.1935			1728			1741.8125	1883.79
31								
33								
36								
38								
40								
43								
45								
47								
50								
52								
54								
57								
59								
61								
64								
66								
68								
71								
73								
75								
78								
