# Supplementary material for: Preliminary evaluation of alpha-emitting radioembolization in animal models of hepatocellular carcinoma
Source: PLoS One. 2022 Jan 21;17(1):e0261982. doi: 10.1371/journal.pone.0261982 (PMC8782514; doi:10.1371/journal.pone.0261982)
Supplement: S5 Table — (PDF) [file pone.0261982.s005.pdf]

Days post-injection	Untreated							
0	37.908	841	103.968	377.196	269.7695	424.128	319.95	161.86
1	38.088	435.896	107.911	818.62	289.338	658.56	415.15	525
2	58.8245	557.7325	75.816	696.192	546.21	654.6375	126.852	469.3
4	87.079	703.8375	188.65	1006.7805	960.6915	1139.0625	371.907	595.50
7	121.086	818.892	208.088	1097.728	978.602	1148.175	210.826	417.15
10	225.108	787.968	355.008	1461.2375	1171.206	1322.464	205.1665	497.31
12	194.5085	686.8085	126.852	1272.384	1285.632	1545.3375	470.596	458.78
15	451.25	795.328	121.0545	1631.7735	1598.7	1431.43	325.125	871.80
17	662.924	888.04	194.0625			1653.9745	468.18	896.62
19	683.1575	1008.2	125.692				465	1294.9
22	844.74	1117.1625	199.874				456.19	925.75
24	1166.948	1645.451	269.001				653.455	1568
26	1239.232		332.682				927.5455	
29	1127.196		517.495				1968.3	
31	1865.15		688.788					
33			742.5625					
36			826.062					
38			1225.25					
40			1646.4					
43								
45								
47								
50								
52								
54								
57								
59								
61								
64								
66								
68								
71								
73								
75								
78								
